# The Role of Iron-Overloaded Macrophages in Mesenchymal Stem Cell Senescence and Anemia in Myelodysplastic Syndromes: Protocol for an In Vitro Study

**DOI:** 10.2196/77936

**Published:** 2026-01-13

**Authors:** Lu Bai, Fan Wang, Yaxian Jiang, Hongmei Ouyang

**Affiliations:** 1 Department of Clinical Laboratory The First People’s Hospital of Yunnan Province (Affiliated Hospital of Kunming University of Science and Technology) Kunming, Yunnan Province China

**Keywords:** myelodysplastic syndromes, mesenchymal stem cells, iron-overloaded macrophages, Keap1-Nrf2 pathway, anemia

## Abstract

**Background:**

Myelodysplastic syndromes (MDSs) are a group of highly heterogeneous myeloid clonal diseases. Anemia is the most common clinical symptom, yet its pathogenesis remains incompletely understood. Preliminary evidence suggests an increase in macrophage infiltration and iron load in the bone marrow of patients with an MDS, alongside elevated interleukin-6 (IL-6) expression in bone marrow mesenchymal stem cells (BMSCs). The Kelch-like ECH-associated protein 1 (Keap1)–nuclear factor erythroid 2–related factor 2 (Nrf2)–antioxidant response element (ARE) pathway is a critical regulator of cellular antioxidant responses and inflammation, but its role in mediating the effects of iron overload in the microenvironment of patients with an MDS remains unclear.

**Objective:**

This study aims to investigate the hypothesis that iron-overloaded bone marrow macrophages promote BMSC senescence and IL-6 secretion via the Keap1-Nrf2-ARE pathway, thereby impairing the survival and differentiation of hematopoietic stem cells and hematopoietic progenitor cells and contributing to anemia. Specific objectives are to (1) quantify the macrophage iron load across major MDS subtypes and correlate it with anemia severity and prognosis; (2) define the phenotype of erythroid island macrophages (CD68+, CD169+, and vascular cell adhesion molecule 1+) and the expression of endothelial microparticles in bone marrow tissues of patients with an MDS; and (3) experimentally test whether iron-overloaded macrophages and their exosomes regulate IL-6 secretion and senescence in BMSCs via the Keap1-Nrf2-ARE pathway.

**Methods:**

Bone marrow samples will be collected from patients with key MDS subtypes (MDS with single-lineage dysplasia, refractory anemia with ring sideroblasts, MDS with multilineage dysplasia, and MDS with excess blasts; n=30 per subgroup) and control patients with iron-deficiency anemia (n=30). Methods will include histochemistry (Perls Prussian blue staining), immunohistochemistry and immunofluorescence (for macrophage and endothelial microparticle analysis), enzyme-linked immunosorbent assay, flow cytometry, quantitative polymerase chain reaction, and Western blotting. An in vitro model of iron-overloaded macrophages will be established using phorbol 12-myristate 13-acetate–differentiated THP-1 cells treated with ferric ammonium citrate. Exosomes will be isolated from these macrophages via ultracentrifugation. The effects of iron-overloaded macrophages and their exosomes on BMSC IL-6 secretion, senescence (senescence-associated β-galactosidase staining), and Keap1-Nrf2-ARE pathway activity will be assessed in coculture systems, with and without pharmacological inhibitors (5,6-dichloro-1-β-D-ribofuranosylbenzimidazole) or activators (dimethyl fumarate) of Nrf2.

**Results:**

The study received ethics approval in December 2024. Patient recruitment and sample collection are in progress. As of December 2025, a total of 85 samples had been accrued. Pilot experiments to optimize macrophage differentiation and iron loading conditions have been completed. The full experimental workflow, including all sample analyses and in vitro experiments, is anticipated to be completed by May 2026.

**Conclusions:**

This study is expected to elucidate a novel molecular mechanism linking iron overload in macrophages to BMSC dysfunction and anemia in MDS. The findings could identify the Keap1-Nrf2-ARE pathway as a potential therapeutic target for managing MDS-related anemia.

**International Registered Report Identifier (IRRID):**

DERR1-10.2196/77936

## Introduction

Myelodysplastic syndromes (MDS) are a group of highly heterogeneous myeloid clonal diseases, characterized by abnormal hematopoietic cell development in one or more myeloid lineages, manifesting as ineffective hematopoiesis and a high risk of transformation into acute myeloid leukemia. Anemia is a common clinical symptom and an important factor affecting the survival and quality of life of patients with MDS [1]. However, the current pathogenesis of anemia in MDS remains unclear.

About 80% to 85% of patients with MDS are anemic [2], and intracellular iron content is increased in erythrocytes. However, studies examining iron levels in bone marrow macrophages are limited. Anemia has been shown to be associated with abnormalities in the hematopoietic microenvironment [3]. The main hematopoietic cells in the hematopoietic microenvironment are macrophages, mesenchymal stem cells (MSCs), adipocytes (fat cells), sinusoidal endothelial cells, and fibroblasts [4,5]. Macrophages not only serve as the core components of the innate immune system but also play a role in phagocytosis, chemotaxis, the secretion of bioactive substances, and the participation and regulation of immune responses. In addition, macrophages are the main iron storage cells and key members of hematopoietic niches. We have completed the culture and identification of macrophages and MSCs and detected the increased infiltration density of CD68+, CD169+, and vascular cell adhesion molecule 1 (VCAM-1)+ macrophages in the bone marrow tissues of patients with MDS. However, the relationship between the intensity of iron staining in macrophages in MDS bone marrow tissue and anemia severity, disease subtype, and disease process requires further clarification. In addition, macrophages within erythroblastic islands provide iron to promote the differentiation and maturation of nucleated erythrocytes at all stages. Moreover, MSCs play an important role in the survival, differentiation, and maturation of hematopoietic stem cells (HSCs) or hematopoietic progenitor cells (HPCs). However, the correlation between iron-overloaded macrophages and their exosomes and the secretion of the inflammatory senescence-associated cytokine interleukin-6 (IL-6) by MSCs remains unclear, and the effects of this interaction on the survival, differentiation, and maturation of HSCs and HPCs remain to be determined.

The hematopoietic microenvironment is the site of survival, differentiation, and maturation of hematopoietic cells. It consists of reticular connective tissue and nonhematopoietic cells and is characterized by alterations in hematopoietic-matrix interactions, abnormal production of growth factors, and dysregulation of hematopoietic growth factor signaling [6-9]. The erythroid hematopoietic island is composed of 1 to 2 macrophages at the center and the surrounding erythrocytes at different stages of differentiation. Under physiological or pathological conditions, the functional state of macrophages can be regulated to affect erythropoiesis [10,11]. During erythroid maturation, macrophages promote the maturation of nucleated erythrocytes into reticulocytes by retaining immature erythrocytes in bone marrow and clearing old red blood cells. In the interaction between macrophages and erythroid nucleated cells, the anatomical structure and functional integrity of erythroid hematopoietic islands are necessary. Intercellular adhesion molecules are important components for maintaining the structural and functional integrity of erythroid hematopoietic islands [12]. Therefore, the phenotype of macrophages within erythroid islands in MDS bone marrow must be clarified, and the relationship between macrophage phenotype and the expression and differentiation of erythroid adhesion molecules requires further investigation.

Given the difficulty in obtaining macrophages from erythroid hematopoietic islands, research on their identification, function, and mechanism of action supporting erythroid development has been limited. Currently, the macrophage markers within erythroid hematopoietic islands include CD68, CD 169, and VCAM-1 [13,14]. CD68 is a canonical marker of human macrophages. CD 169, also known as sialoadhesin (Siglec-1), belongs to the sialic acid–binding immunoglobulin-like lectin family and is expressed in specific macrophage subsets. VCAM-1 is a vascular cell adhesion molecule that belongs to the immunoglobulin superfamily and assists in the homing of HSCs and HPCs. Li [14] demonstrated functional heterogeneity among erythroid island macrophages and identified at least 4 macrophage subgroups in the human fetal liver, including populations associated with early erythrocytes, late erythrocytes, and erythroblast nuclear phagocytosis [15,16]. However, the identification and functional analysis of macrophages within erythroid hematopoietic islands in the anemic bone marrow of patients with MDS are rare. On the basis of the existing clinical and laboratory resources, confocal immunofluorescence microscopy was proposed to analyze the expression of CD68+, CD 169+, and VCAM-1+ macrophages in the bone marrow tissues from included study samples and to preliminarily determine the phenotype and frequency of macrophages in the erythropoiesis island of bone marrow.

Molecules adhering to macrophages that interact with nucleated erythrocytes play an important role in promoting the differentiation and maturation of nucleated erythrocytes. According to Mao et al [15], endothelial microparticles (EMPs) were closely associated with the formation of hematopoietic islands [17,18]. EMP antagonists can inhibit the proliferation, maturation, and enucleation of erythrocytes and greatly reduce the number of erythrocytes. EMP-knockout mice showed severe anemia. However, the expression of EMP in bone marrow tissue and its relationship with anemia and clinical characteristics remain unclear. Therefore, this study aims to detect the expression of EMP in bone marrow tissues and determine the clinical significance of EMP expression in anemia of MDS.

The exosome is the carrier of intercellular communication within the liquid microenvironment of the body and is a nanoscale extracellular vesicle secreted by living cells. It carries biological information molecules such as proteins, nucleic acids (mRNA, microRNA, DNA, etc) and lipids derived from parent cells, and it can regulate the physiological and pathological processes in target cells over short or long distances through body fluids [19]. Iron is not only an important raw material in erythropoiesis but also a key catalyst for redox reactions. It affects bone marrow hematopoiesis by modulating the level of oxidative stress in vivo [20-22]. However, the molecular mechanism by which the Kelch-like ECH-associated protein-1 (Keap1)–nuclear factor erythroid-2–related factor 2 (Nrf2)–antioxidant response element (ARE) signaling pathway mediates the effects of iron-overloaded macrophages and their exosomes on inflammatory cytokine IL-6 secretion and cellular senescence remains unclear.

The Keap1-Nrf2-ARE pathway plays a critical role in antioxidant responses, modulation of inflammation, and regulation of the immune balance of Th1/Th2 [23]. Nrf2 is the core transcription factor of an endogenous antioxidant response that regulates apoptosis, cell cycle, inflammatory cytokines, and immune response to induce tumor, inflammation, and aging-related diseases [24,25]. After the activation of the Keap1-Nrf2-ARE pathway, the expression of various downstream target proteins is upregulated, such as the antioxidant proteins and enzymes heme oxygenase 1, superoxide dismutase, glutathione peroxidase, and γ-glutamylcysteine synthetase. Iron overload was defined as serum ferritin levels greater than 1000 μg/L. Abnormal activation of the AMP-activated protein kinase-mitochondrial fission factor-dynamin-related protein 1, Wnt/β-Catenin, and reactive oxygen species-hypoxia-inducible factor 1α pathways has been reported to affect the secretion of cytokines and immunomodulatory functions of bone marrow MSCs (BMSCs) [19,26]. However, whether iron-overloaded macrophages and their exosomes regulate the oxidative stress and inflammatory state of BMSCs through the Keap1-Nrf2-ARE pathway remains unclear.

On the basis of the preliminary work of our team, we hypothesize that bone marrow iron-overloaded macrophages in patients with anemia regulate IL-6 secretion and cellular senescence through the Keap1-Nrf2-ARE pathway, thereby influencing the survival and differentiation of HSCs and HPCs and contributing to anemia [27-32]. We have completed the culture and identification of macrophages and MSCs, and we observed an increase in the infiltration density of macrophages and iron overload in bone marrow tissues [33]. MDS are highly heterogeneous diseases that are divided into 7 subtypes: MDS with single-lineage dysplasia, refractory anemia with ring sideroblasts (RARS), MDS with multilineage dysplasia, MDS with excess blasts, MDS unclassifiable, 5q syndrome, and refractory cytopenia of childhood. On the basis of the large sample size of MDS with subtypes including MDS with single-lineage dysplasia, RARS, MDS with multilineage dysplasia, and MDS with excess blasts, this study primarily aims to detect the iron load of bone marrow macrophages of these 4 MDS subtypes; analyze the correlation between iron load and MDS classification, anemia severity, prognosis, and other clinical and laboratory data; and determine the clinical significance of the macrophage iron overload of MDS bone marrow. In addition, the subtypes of erythropoietic macrophages and the expression of EMP in MDS bone marrow tissues will be determined. Furthermore, this study will investigate whether iron-overloaded macrophages and their exosomes regulate the expression of IL-6 and the senescence of MSCs through the Keap1-Nrf2-ARE pathway, thereby influencing the survival and differentiation of HSCs and HPCs and contributing to the occurrence and development of anemia.

This study aims to explore the clinical significance of iron overload in bone marrow macrophages by histochemistry, immunohistochemistry, enzyme-linked immunosorbent assay, flow cytometry, quantitative polymerase chain reaction, and Western blotting to determine the phenotype and function of erythroid hematopoietic island macrophages and to assess whether iron-overloaded macrophages upregulate the expression level of MSC-derived IL-6 via the Keap1-Nrf2-ARE pathway and promote cellular senescence, thereby affecting the survival and differentiation of HSCs and HPCs.

## Methods

### Design

The study design will be an in vitro cell biology study.

### Time and Setting

This study will be performed at the First People’s Hospital of Yunnan Province. The research is currently underway. Samples will be stored at −80 °C, and laboratory analyses will be performed every 2 weeks.

### Ethical Considerations

This study was approved by the Clinical Research Ethics Committee of the First People’s Hospital of Yunnan Province (KHLL2021-188). Written informed consent will be obtained from all participants before sample collection. All participant data and samples will be deidentified and coded to ensure confidentiality. Data will be stored on a secure, password-protected server. No participant compensation will be provided.

### Participant Recruitment and Samples

Bone marrow biopsy specimens will be collected from patients diagnosed with MDS (including the following World Health Organization criteria subtypes: MDS with single-lineage dysplasia, RARS, MDS with multilineage dysplasia, and MDS with excess blasts) and control patients with iron-deficiency anemia (IDA). Inclusion criteria for the MDS group will be a confirmed diagnosis by a hematologist, while exclusion criteria may include prior allogeneic stem cell transplantation or concurrent active malignancy. The control group will consist of patients with confirmed IDA. A target sample size of 30 per subgroup was estimated based on practical feasibility and preliminary data variability from previous similar studies. Clinically relevant information, including complete blood counts, serum ferritin, transfusion history, and cytogenetic data (eg, mutations in tet methylcytosine dioxygenase 2, additional sex combs like transcriptional regulator 1, and tumor protein p53), will be collected.

### Experimental Procedure

#### Expression Intensity of Iron-Carrying Macrophages in Bone Marrow Tissues Determined by Prussian Blue Staining

Prussian blue staining is a classical histochemical reaction, used for the sensitive detection of ferric iron in tissue and is routinely performed in the laboratory. For semiquantitative analysis, the staining results will be independently evaluated by 2 experienced examiners.

#### Immunohistochemistry and Immunofluorescence Analysis

Immunofluorescence multiplex staining and immunohistochemistry were used to detect the expression of erythroid hematopoietic island CD68+, CD169 +, and VCAM-1+ macrophages and EMPs in the bone marrow tissues from the included study samples.

The paraffin-embedded sections of conventional tissue specimens will be prepared using standard processing procedures. The specific primary antibody and its corresponding secondary antibody will be selected to optimize the working concentration for multiple staining. Positive cell density will be quantified using image analysis software (eg, QuPath).

#### Establishment and Identification of Iron-Overloaded Macrophages In Vitro

##### Cell Culture

Resuscitated and expanded THP-1 cells will be cultured in RPMI-1640 complete medium containing 10% fetal bovine serum.

##### Induction of Macrophage Differentiation

THP-1 cells will be induced to differentiate into macrophages using 50 μg/mL of phorbol 12-myristate 13-acetate (PMA) for 24 hours and will be cultured in an RPMI-1640 complete medium without exosomes. Differentiation will be confirmed by flow cytometry analysis of surface markers CD11b and CD14.

##### Construction of Iron-Overloaded Macrophages

To construct iron-overloaded macrophages, multiple concentrations of ferric ammonium citrate (FAC; 10, 20, 40, 80, and 160 μmol/L) will be applied to PMA-induced THP-1 cells and cultured for 24 hours. The control group (THP-1+PMA) will receive equivalent volumes of phosphate-buffered saline (PBS). After the FAC treatment, cell viability will be assessed using the Cell Counting Kit-8 assay with absorbance measured at 450 nm. By comparing the optical density 450 values between the FAC treatment group and the control group, the effect of FAC concentration on cell viability will be analyzed, and the appropriate FAC concentration for constructing an iron overload model will be determined.

##### Model Validation

To verify the successful construction of the model, different concentrations of FAC (10, 20, 40, 80, and 160 μmol/L) will be added to PMA-induced M0 macrophages with THP-1 will be continued for 24 hours. After treatment, the Calcein-AM fluorescent probe will be added, and after incubation for a period, intracellular fluorescence intensity will be observed and recorded using a fluorescence microscope. Changes in fluorescence intensity will reflect alterations in the intracellular labile iron pool, confirming successful iron overload model establishment.

#### Extraction and Identification of Exosomes From Iron-Overloaded Macrophages and Culture and Identification of BMSCs

Our research group has established an experimental method for extracting exosomes by ultracentrifugation and using the SBI kit (ExoAb Antibody Kit, Shanghai Yanhui Biotechnology Co, Ltd). BMSCs will be extracted from bone marrow samples of patients with IDA based on our previous work [33].

#### BMSC Isolation and Primary Culture

Bone marrow mononuclear cells will be isolated from bone marrow aspirates obtained from patients with IDA (control group) using density gradient centrifugation. Briefly, bone marrow samples will be diluted with PBS and carefully layered over Ficoll-Paque PLUS (Cytiva). After centrifugation at 400 g for 30 minutes at room temperature, the bone marrow mononuclear cells layer at the interface will be collected, washed twice with PBS, and counted. The cells will be resuspended in complete culture medium consisting of α-minimum essential medium supplemented with 10% fetal bovine serum, 100 U/mL penicillin, and 100 μg/mL streptomycin. Cells will be seeded into culture flasks at a density of 1 to 2×10^5^ cells/cm² and maintained at 37 °C in a humidified atmosphere of 5% CO_2_. The medium will be changed every 3 to 4 days to remove nonadherent cells.

#### BMSC Identification by Flow Cytometry

The immunophenotype of the cultured BMSCs will be confirmed by flow cytometry. Briefly, passage 3 BMSCs will be harvested, washed, and incubated with fluorescein-conjugated antibodies against classic mesenchymal surface markers (eg, CD73, CD90, and CD105) and hematopoietic lineage markers (eg, CD34, CD45, and HLA-DR). Appropriate isotype controls will be used. A minimum of 10,000 events will be acquired using a flow cytometer (eg, BD FACSCanto II; BD Biosciences), and data will be analyzed using FlowJo software (BD Biosciences). BMSCs will be defined as positive for CD73, CD90, and CD105 (>95% positive) and negative for CD34, CD45, and HLA-DR (<2% positive).

#### Coculture of Iron-Overloaded Macrophages and Their Exosomes With BMSCs

A transwell coculture system will be used. The coculture model of iron-overloaded macrophages and their exosomes will be established with BMSCs, and the proportion of senescent cells among BMSCs will be detected by senescence-associated β-galactosidase staining. The expression of IL-6 will be detected by enzyme-linked immunosorbent assay or flow cytometry.

#### Gene and Protein Expression Analysis

Quantitative polymerase chain reaction will be used to detect the expression of Keap1, Nrf2 and ARE mRNA in BMSCs, as well as the intervention effect of the Nrf2 inhibitor 5,6-Dichloro-1-β-D-ribofuranosylbenzimidazole or activator dimethyl fumarate. A semiquantitative analysis of the β-actin gene will also be performed.

Western blot will be used to detect the expression of Nrf2 in BMSCs. The semiquantitative analysis will be performed using β-actin as the internal control protein. The Western blot experiment is a routine experimental process of the laboratory. The antibody will be determined by the protein expressed by the selected target gene. Proteins will be extracted using the radioimmunoprecipitation assay buffer kit and protein will be separated by 10% sodium dodecyl sulfate-polyacrylamide gel electrophoresis. The experimental conditions will be optimized by changing the transmembrane conditions, the type and time of blocking reagent, and the concentration and incubation time of primary and secondary antibodies.

#### Isolation and Cultivation of HSCs and HPCs

HSC and HPC samples extracted from umbilical cord blood obtained from healthy term deliveries will be used to evaluate the effects of iron-overloaded macrophages on the survival and differentiation of HSCs and HPCs by flow cytometry and soft agar assays.

### Main Observation Indicators

The basic morphology of bone marrow, the iron-staining intensity of macrophages, the phenotype of macrophages in erythroid hematopoietic islands, the expression of IL-6 in bone marrow, the expression of IL-6 in BMSCs, and the effects of iron-overloaded macrophages on the survival and differentiation of HSCs and HPCs will be observed in this study.

### Statistical Analysis

Data analysis will be performed using SPSS software (version 26.0; IBM Corp). Descriptive data will be presented as mean (SD). Normality will be tested using the Shapiro-Wilk test. Comparisons between 2 groups will be performed using the Student *t* test (2-tailed; parametric) or Mann-Whitney *U* test (nonparametric). Comparisons among multiple groups will be performed using 1-way ANOVA with post hoc tests or the Kruskal-Wallis test. A *P* value <.05 will be considered statistically significant.

## Results

Ethical approval was secured in December 2024. Patient recruitment and sample collection are underway. To date, pilot experiments have successfully established the protocols for THP-1 macrophage differentiation, FAC-induced iron loading, and BMSC culture. The main phase of sample processing and in vitro validation experiments is in progress. The timeline for the study anticipates completion of data collection by May 2026, followed by data analysis and manuscript preparation. A schematic timeline is presented in Table 1.

**Table 1:** Study timeline.

Activity	Start date	End date	Status
Ethics and study setup	December 2024	February 2025	Completed
Patient recruitment and sample collection	January 2025	February 2026	Ongoing
Pilot experiments (method optimization)	December 2024	March 2025	Completed
Main laboratory analysis	April 2025	May 2026	Ongoing
Data analysis	May 2026	June 2026	Planned
Manuscript preparation	June 2026	July 2026	Planned

## Discussion

### Anticipated Findings

We anticipate that this study will demonstrate a significant correlation between bone marrow macrophage iron load and the severity of anemia in MDS. Furthermore, we expect to show that iron-overloaded macrophages and their exosomes can induce cellular senescence and IL-6 secretion in BMSCs, and that this effect is mediated, at least in part, through the Keap1-Nrf2-ARE signaling pathway.

### Comparison With Prior Work

Our previous work showed increased macrophage infiltration in MDS [33], consistent with other reports of microenvironment inflammation [3]. This study builds on that by focusing specifically on the iron content of these macrophages and its functional consequences. The link between iron overload and oxidative stress is well established [10], but its specific effect on the Keap1-Nrf2 axis in the context of BMSCs in MDS is novel. Our findings are expected to complement recent research on mitochondrial dysfunction in MSCs from patients with MDS [16].

### Strengths and Limitations

In this study, the iron load of macrophages in the bone marrow microenvironment will be taken as the starting point to determine whether iron-overloaded macrophages promote the IL-6 expression in BMSCs and whether cellular senescence affects HSC survival and differentiation, thereby providing a new perspective for exploring the pathogenesis of different types of anemia. Elucidating the molecular mechanisms of MDS-related anemia and providing a theoretical basis for making diagnostic and treatment strategies are important to supplement and enrich the information of macrophage iron load in the bone marrow microenvironment, clarify the phenotype and frequency of macrophages in the erythroid hematopoietic islands of bone marrow, assess the effect of EMP expression on erythroid maturation and differentiation, determine the clinical significance of the iron load of macrophages, and confirm the molecular mechanism by which iron-overloaded macrophages promote IL-6 secretion and cellular senescence in BMSC.

A limitation, as noted by a reviewer, is the use of patients with IDA as the sole control group, which does not allow for the separation of the effects of anemia from those of iron status. While including an iron-deficient nonanemic control would be ideal, obtaining bone marrow samples from such individuals is ethically challenging. This limitation will be acknowledged, and the in vitro models will be critical for dissecting the specific role of iron.

In addition, this study has a limited sample size because of time and budget constraints. In the future, the sample size should be expanded to explore the diagnosis and treatment strategies of MDS-related anemia in different countries and races.

### Future Directions and Dissemination

If our hypothesis is confirmed, future work will focus on validating these findings in animal models and exploring therapeutic interventions targeting the Keap1-Nrf2 pathway in MDS. The results of this study will be disseminated through publication in peer-reviewed scientific journals and presentations at international conferences.

## Abbreviations

ARE: antioxidant response element
BMSC: bone marrow mesenchymal stem cell
EMP: endothelial microparticle
FAC: ferric ammonium citrate
HPC: hematopoietic progenitor cell
HSC: hematopoietic stem cell
IDA: iron-deficiency anemia
IL-6: interleukin-6
Keap1: Kelch-like ECH-associated protein 1
MDS: myelodysplastic syndromes
MSC: mesenchymal stem cell
Nrf2: nuclear factor erythroid 2–related factor 2
PBS: phosphate-buffered saline
PMA: phorbol 12-myristate 13-acetate
RARS: refractory anemia with ring sideroblasts
VCAM-1: vascular cell adhesion molecule 1

## Data Availability

The datasets generated or analyzed during this study will be available from the corresponding author on reasonable request.
